# *Publisher's Note* added on 29 June 2006 

**DOI:** 10.3390/11040232

**Published:** 2006-06-29

**Authors:** Shu-Kun Lin

There is a pagination error in this paper. The page number for the first page (Sergei V. Trepalin *et al*. *Molecules* 2006, *11*, 219) is assigned correctly. However, the following pages were not correctly paginated and the final page number should be 231 instead of 241. The original file is still provided. Pages 232-241 are thus taken as blank pages. We apologize for this error and for any inconvenience.

